# Simulated-based training for ultrasound-guided popliteal sciatic nerve block: determining the learning curve and transference to real patient

**DOI:** 10.1186/s41077-025-00389-5

**Published:** 2025-11-11

**Authors:** Pablo F. Miranda, Andrea L. Araneda, Natalia P. Molina, Felipe G. Miranda, Christopher Morrison, Marcia A. Corvetto, Fernando R. Altermatt

**Affiliations:** 1 División de Anestesiología, Escuela de Medicina, Pontificia Universidad Católica de Chile, Marcoleta 367, Santiago, 8330024 Chile; 2 Experimental Surgery and Simulation Center, Facultad de Medicina, Pontificia Universidad Católica de Chile, Santiago, Chile

**Keywords:** Simulation training, Learning curve, Anesthesiology/education, Peripheral nerve block

## Abstract

**Background:**

The following study aims to determine the learning curve experienced by anesthesia residents when training for an ultrasound-guided popliteal sciatic block and the transference of this training to real patient situations.

**Methods:**

After approval by the ethics committee, eleven first-year anesthesia residents were recruited to participate in a simulation-based training program to perform a single shot in plane popliteal sciatic block. Training consisted of 10 individual sessions, with direct feedback from the instructor, with a specific Laerdal® popliteal sciatic block phantom, lasting one hour and distributed weekly. At the end of each session, the resident’s performance was assessed. Residents were videotaped while performing the block, which was to be evaluated using a validated global rating scale (GRS). Additionally, a tracking motion device attached to the operator's hands (Imperial College Surgical Assessment Device, ICSAD) recorded the total distance traveled by both hands (Total path length, TPL), number of movements (NM), and total procedure time (TPT). One week later, the same assessment was done on a real patient.

**Results:**

Ten residents completed the training and the assessments. Median values of GRS scores significantly improved from 15 to 28.3 through the training (*p* = 0.006). Regarding ICSAD scores, TPT improved from 126 to 63.4 s (*p* = 0.002), and TPL improved from 11.07 to 9.4 m (*p* = 0.322). When comparing the last simulated session and the subsequent measurement in an actual patient, median values of GRS, TPL and NM were not different.

**Conclusions:**

This simulation-based training program significantly improved residents’ proficiency in an ultrasound-guided popliteal sciatic block. The learning curve plateaued at session 7, and this improvement was transferred to the real patient setting.

As expected, residents needed more time for the first block on a real patient than for the last simulated session.

**Clinical trial number:**

ClinicalTrials.gov, identifier NCT06081790.

## Introduction

Peripheral nerve blocks (PNB) are a type of regional anesthesia that consists of an injection of local anesthetics near a nerve or group of nerves, mainly used to alleviate pain in the perioperative context [1]. Benefits of PNB include the decrease of adverse effects to opioids, better pain management compared to systemic analgesia, better sleep quality, and the possibility of earlier hospital discharge of patients [2]. Ultrasound-guided PNB facilitates the direct visualization of nerves and anatomic structures, the correct placement of the needle and the distribution of local anesthesia. As with all procedures, it is not exempt from risks and requires adequate training of practitioners to ensure safe performance on patients.

Simulation-based training has proven to be a teaching strategy that improves learning, provides practice opportunities and adequate feedback and allows practitioners to develop proper clinical competencies [3]. A systemic review and metanalysis regarding educational methods used to teach invasive medical procedures, [4] reveals that simulation-based training is the most effective methodology when teaching these procedures. Specifically in anesthesia procedures, simulation-based training has proven to be effective when practicing lumbar punctions, central venous catheters insertions and peripheral central catheter insertions [5–7].

Simulation-based training for ultrasound-guided regional anesthesia has emerged as an effective and efficient teaching method for trainees [8]. Acquiring skills in regional anesthesia, including ultrasonography, has become a mandatory component in anesthesiology specialist’s training [9].

Regarding the learning curve of peripheral nerve block technique, there is currently some research available. Kim et al. described an improvement in the quality of injections in a simple phantom, within 5 attempts for unexperienced students [10]. Areti et al. mapped the learning curve of anesthesiology residents who scanned volunteers to identify structures of the branchial plexus, requiring 7 sessions [11]. Finally, Kollmann et al. described students’ learning CUSUM curves for ultrasound-guided continuous femoral nerve block on real patients, concluding that they needed 12 attempts [12].

In this context, little data is available on the learning process of ultrasound-guided peripheral nerve blocks. Today, with current advances in the field of simulation training, we should have learning curves for each procedure and evidence of the transference of these skills learned in a simulated environment to a real patient situation [13]. We believe that having objective data in the description of learning curves during simulated-based training will let us know precisely how many sessions are necessary to train residents without experience in regional anesthesia before performing this procedure on a real patient.

This study aimed to develop a simulation-based program for novice anesthesia residents, to develop skills to perform an ultrasound-guided popliteal block adequately. Additionally, we intended to construct a learning curve for this procedure to determine the number of training sessions necessary to learn the procedure. Finally, we aimed to measure the transference of learned skills in the simulated environment to the real clinical experience after training.

## Methods

### Study design

This was a quasi-experimental prospective cohort study, in which the intervention was a simulated-based training program to acquire skills to perform a single shot ultrasound guided in plane popliteal block.

### Participants design

The institutional ethics committee approved this report and requested the need for informed consent, approval number 230316005. (Comité de Ética en Investigación, Facultad de Medicina, Pontificia Universidad Católica de Chile). Additionally, the protocol was registered in ClinicalTrials.gov, identifier NCT06081790.

All first-year anesthesia residents received an invitation email explaining the training protocol. Residents could voluntarily agree to participate or not. Residents who had previously performed more than 3 peripheral nerve blocks under ultrasound in a real patient were excluded. Once eligibility was assessed, a second email describing the protocol was sent. Finally, 11 residents signed informed consent and were recruited for the study.

### Educational support material

Educational material including theoretical and practical concepts on ultrasound guided techniques and popliteal block were delivered. The e-learning platform C1DO1 (www.c1do1.ai) was used for the online delivery of educational material.

Residents were asked to review the educational material prior to the beginning of the protocol (assessments and training sessions) to have a homogeneous theoretical framework among the participants. This educational material was delivered in 2 recorded lectures and 4 short video capsules. Residents had access to the instructional material through the online C1DO1 platform at anytime from anywhere using their electronic devices and internet access during the protocol.

### Intervention

Simulated training consisted of 10 individual sessions in the simulation center, lasting one hour and distributed weekly. Residents were scheduled to practice with the instructor in a one-to-one session. An instructor was present throughout the entire session and gave instructions and direct feedback in real-time throughout all sessions (traditional face-to-face feedback modality). Instructors for feedback were 2 anesthesiologists with a regional anesthesia fellowship, experts in simulation and feedback (F.M and P.M.). During the session, both scanning and puncture skills were worked on to perform a single shot ultrasound guided in plane popliteal sciatic block. Instructors were handed a roadmap with 7 specific goals for the practical sessions to facilitate and standardize instruction:

1. Preparation for the procedure.
2. Steps of the procedure.
3. Instruments and how to handle them.
4. Ultrasound and transducer set up.
5. Scanning techniques and imaging acquisition.
6. Eye hand coordination.
7. Needle visualization and manipulation.

Instructors aimed to reach all 7 goals during each session. However, depending on every participant’s capability, goals were adjusted and incorporated progressively during the sessions. The structure of the session consisted of 45 min for practice and 15 min for assessment.

A basic puncture phantom and a specific sciatic popliteal block phantom (Blue Phantom®, Redmond, WA) were used. The materials used to perform the block were an echogenic Stimuplex Ultra 360™ 20 Gauge and 4 Inch insulated single shot needle (BBraun Stimuplex®) and a Sonus SL-3C Wireless Linear Ultrasound.

### Assessments

After reviewing the educational support material and before the training sessions, all residents performed a pre-training knowledge test, consisted of 12 multiple choice questions.

At the end of each session, during the 10-session training program, an assessment of resident’s performance was done. All the assessments were videotaped for evaluation by two independent and blinded reviewers. The simulation center staff recorded the assessment videos for a standard vision of the participant's hands and the ultrasound image. Two reviewers (A.A. and N.M.) rated each resident’s performance using a global rating scale (GRS) previously validated to assess an ultrasound-guided supraclavicular block [14]. Both were anesthesiologist experts in simulation in healthcare and feedback. During the video assessment, reviewers were blinded to the identity of the resident and the corresponding session number. These 2 reviewers were different from the instructors who gave feedback during the sessions. The GRS assessed general behaviors (time and motion, flow of the procedure) and consisted of 7 items, each rated on a behaviorally anchored 5-point scale, with a maximum total score of 35 (supplementary material 1).

Additionally, two sensors of a tracking motion device were attached to the operator's hands, during the assessment. The Imperial College Surgical Assessment Device (ICSAD) is a device that tracks hand motion during a procedure using sensors placed on the back of the operator’s hands. The total path length of both hands provides an effective index of technical skill and economy of movements during a manual task [15]. In this study, the ICSAD device recorded the total distance traveled by both hands (TPL), number of movements (NM) and total procedure time (TPT). The simulation center staff installed the device in the operators’ hands and recorded the data.

Once the 10-session training program was finished the same assessment was done in a real patient situation, to measure the transference of learning achieved in the simulation environment to real patients. This assessment was done during a period of 2 weeks. With this purpose, residents performed an in plane popliteal sciatic block in recovery room in a patient in prone position, after a foot and ankle surgery. A hospital staff anesthesiologist in charge of the case, joined the resident in the PACU during the procedure, to assure patient safety and the adequate performance of the protocol. They are allowed to halt the procedure if necessary. No feedback was allowed. ICSAD sensors were attached to resident’s hands, below the gloves, to maintain the sterile technique. During the procedure, a video was recorded with the same methodology described above. The simulation center staff were responsible of ICSAD and video records. Later, two blinded reviewers observed the videos, to rate resident’s performance during the block using the same GRS (A.A. and N.M.). These two blinded reviewers assessed all the videos (training sessions and real patient situation). As stated above, video reviewers and instructors for feedback were different.

Blocks were performed using an echogenic Stimuplex Ultra 360™ 20-gauge, 4-inch insulated single-shot needle (B. Braun Stimuplex®) in conjunction with a Sonosite M-Turbo US machine with an L38 × 10–5 MHz, 38-mm broadband linear array probe (Sonosite Inc, Bothell, Washington).

Finally, an online satisfaction survey was sent to the residents to assess their perceptions after they finished the program. Satisfaction survey consisted in twenty 5-item Likert questions, around the achievement of objectives, the educational material, the platform, the duration of training, the duration of simulation sessions, instructors’ feedback and satisfaction with the training program.

### Sample size

A required sample size of 10 subjects was calculated a priori to detect a difference in the primary outcome (GRS score); the assumed effect size was 1.5 (Cohen's d), and the study power was 0.8 for paired samples (before-and-after training difference). The effect size was calculated based on the improvement in the GRS score of previous simulated trainings of procedural skills [16].

### Statistical analysis

The data were analyzed using SPSS version 15.0 software (Chicago, IL, USA). A non-parametric data distribution was also assumed. GRS and ICSAD values were expressed as median and inter-quarter range. The Wilcoxon signed rank methods were used for the analysis. The intraclass correlation coefficient (ICC) was calculated for interobserver agreement. A *p*-value of 0.05 was considered significant.

## Results

Ten residents completed the 10-session training program and the assessments in a simulated environment and with a real patient. One resident halted the residency program due personal issues, after session 1.

Table 1 presents the demographic characteristics and previous peripheral nerve block experience of residents. All the participants were anesthesia first-year residents. Residents had little experience performing peripheral nerve blocks. Six had performed between 1 and 3 blocks during their residency, and four had not. None of them had previous ultrasound-simulated-based training.

**Table 1:** Demographic data

	***N (%) or Median (IQR)***
**Age**	30.5 (28–31)
**Sex**	
* Male*	4 (40)
* Female*	6 (60)
**Year of Residency**	
* PGY-1*	10 (100)
* PGY-2*	0
* PGY-3*	0
**Previous PNB**	
* 0 blocks*	4 (40)
* 1–3 blocks*	6 (60)
> *3 blocks*	0
**Previous SBT**	
* Intravenous Access*	0
* Central Venous Catheter*	0
* PNB*	0

*IQR* Interquartile Range, *SBT* Simulation-based Training, *PNB* Peripheral Nerve Block

Ten residents answered the pre-training knowledge test, with a median score of 10.8 points out of 12.

**Fig. 1:**
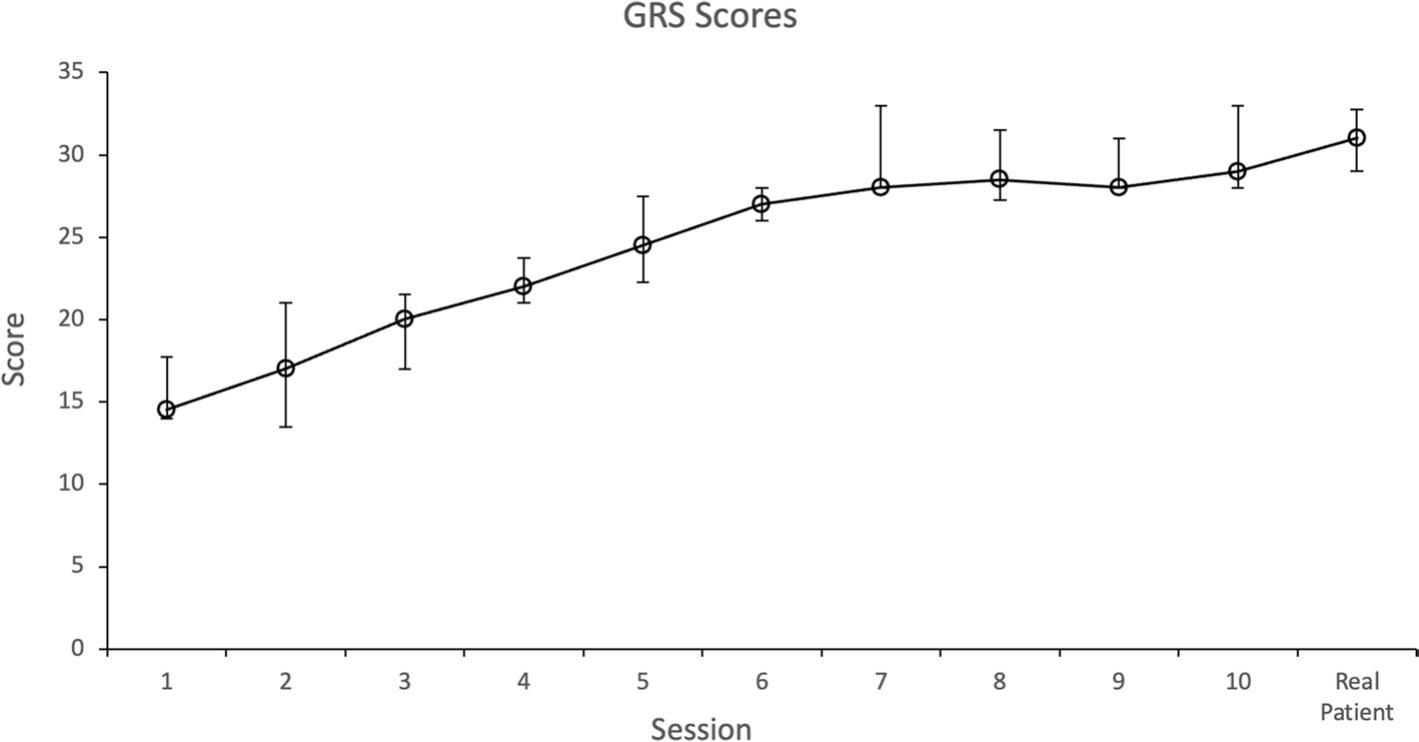
Global Rating Scale (GRS) score of all the residents through the 10 sessions and the transfer session. Data are presented as median value with interquartile ranges for each session

The inter-rater reliability of the observers’ video GRS scores was good to excellent, with an ICC of 0.91 (confidence interval, 0.63–0.98). Median values of GRS scores significantly improved from 15 to 28.3 through the training (*p* = 0.006). Figure 1 shows the progression of GRS scores of all the residents through the 10 sessions and the calculated median value for each session. The learning curve plateaued at session 7, with a GRS median score of 28 points (80% of the total GRS score).

**Fig. 2:**
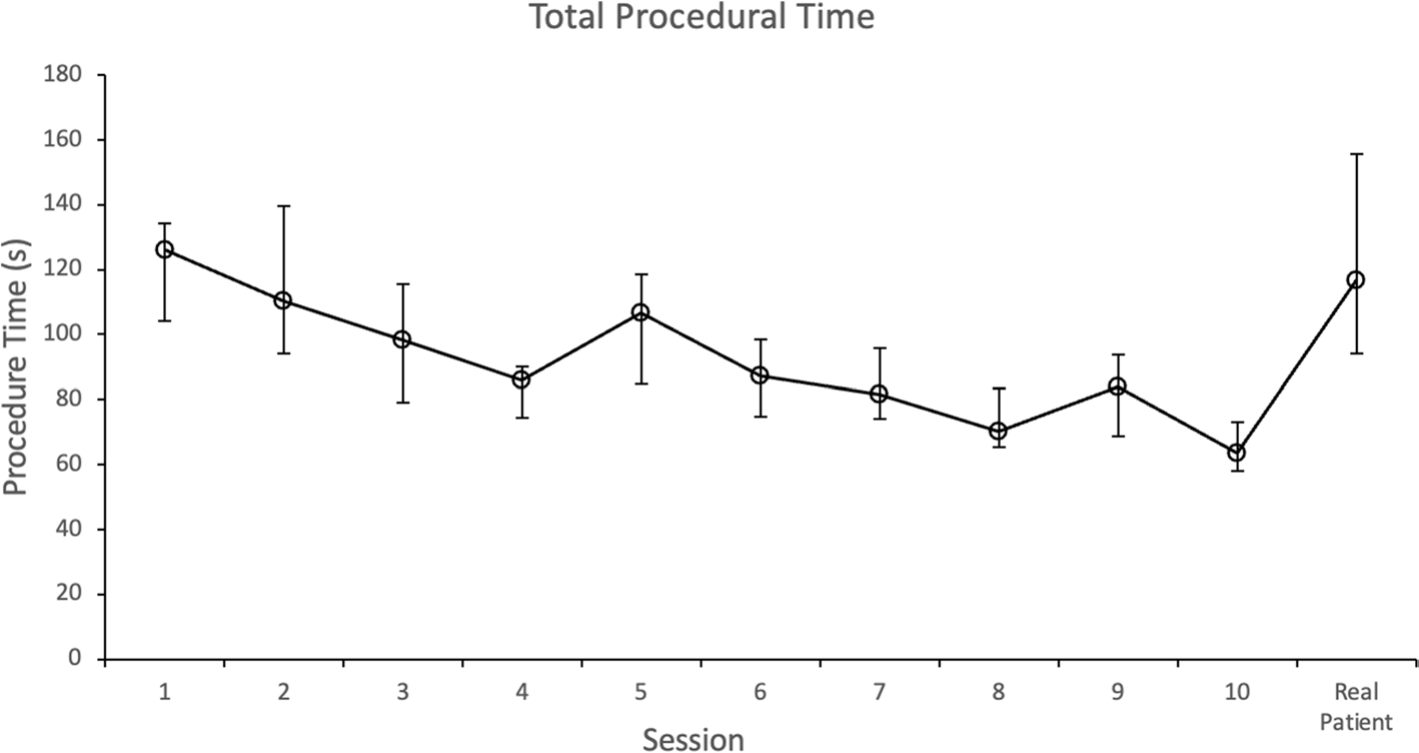
Total procedural time (TPT), measured in seconds, of all the residents through the 10 sessions and the transfer session. Data are presented as median value with interquartile ranges for each session

**Fig. 3:**
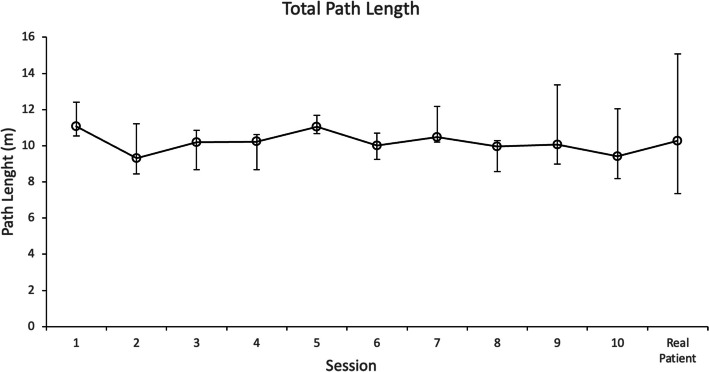
Total path length (TPL), measured in meters, of all the residents through the 10 sessions and the transfer session. Data are presented as median value with interquartile ranges for each session

Results measured with the ICSAD device, including total path length, procedural time, and number of movements, are shown in Table 2. Total procedure time (TPT) decreased significantly from 126 to 63.4 s (*p* = 0.02). The total distance traveled by both hands (TPL) improved from 11.07 to 9.4 m (*p* = 0.322), and the number of movements (NM) from 48 to 41 (*p* = 0.262), both dexterities were not significant. Figures 2 and 3 show the progression of TPT and TPL of all the residents through the ten respectively, sessions and the calculated median value of each session.

**Table 2:** Comparison between Session 1 and Session 10 assessments

	**Session 1**	**Session 10**	***P* value**
GRS scores	15 (14.6–16.1)	28.3 (25.6–29.9)	*p* = 0.006
TPT (s)	126 (104.1–134.2)	63.4 (58,2–73)	*p* = 0.002
NM	48 (42–54)	41.5 (32.8–56.3)	*p* = 0.262
TPL (m)	11.07 (10.6–12.4)	9.4 (8.2–12)	*p* = 0.322

Data are summarized as median and interquartile range

*GRS* Global rating scale scores, *TPT* Total procedural time, *NM* Number of movements, *TPL* Total path length

*P* values obtained when comparing with Wilcoxon signed-rank test

Regarding the assessment in a real patient situation, all the residents finished the procedure successfully in the PACU unit. Comparing the last simulated session (the 10th session) and the subsequent measurement in an actual patient, the median values of global rating scale (GRS), total path length (TPL) and number of movements (NM) were not different (Table 3). Nevertheless, residents needed more time for the first block on a real patient than in the last simulated session (116.6 versus 63.4 s), a statistically significant difference.

**Table 3:** Comparison between Session 10 and Transference assessments

	**Session 10**	**Transference**	***P* value**
GRS scores	28.3 (25.6–29.9)	26.5 (25.3–29.5)	*p* = 0.798
TPT (s)	63.4 (58.2–73)	116.6 (94–155.5)	*p* = 0.02
NM	41.5 (32.8–56.2)	48.5 (39.3–70.8)	*p* = 0.432
TPL (m)	9.4 (8.2–12)	10.3 (7.4–15.1)	*p* = 0.922

Data are summarized as median and interquartile range

*GRS* Global rating scale, *TPT* Total procedural time, *NM* Number of movements, *TPL* Total path length

*P* values obtained when comparing with Wilcoxon signed-rank test

*P* value is considered statistically significant when < 0.05

Finally, the residents' satisfaction survey showed high satisfaction levels with the training program. All residents agreed that the program's objective was clear (100% Likert 5), the course contents were appropriate (100% Likert 5), adequate training duration (100% Likert 5), the platform was appropriate (100% Likert 5) and instructor’s feedback was fitting and centered on the learning objectives (80% Likert 5, 20% Likert 4).

## Discussion

This study demonstrated a significant improvement in novice anesthesia residents’ skills in performing an ultrasound-guided single-shot popliteal sciatic block after a simulation-based program. These results shown a sustained increase of residents’ proficiency scores until session 7, with a subsequent plateau effect. In simulation-based training, the inflection point on the learning curve marks the transition from rapid initial progress to a phase of slower, more complex skill acquisition. At this stage, further improvement requires greater effort, critical thinking, attention to detail, and self-reflection. Recognizing this threshold allows educators to adjust scenario difficulty, enhance feedback, and maintain learner motivation. Rather than representing a barrier, the inflection point is a crucial opportunity to deepen competence and advance toward expert clinical performance. It highlights the shift from basic proficiency to the refinement of integrated, high-level skills essential for real-world practice [17]. With this instructional methodology, we could state that 7 is the number of sessions to achieve the 80% of the proficiency score to perform a popliteal single shot block. Previous studies have been stablished the 80% as the minimum passing score for objective structured assessment of technical skills (OSATS) [18]. It is important to highlight that this improvement was under specific training conditions: 10 sessions lasting 1 h, distributed weekly, with direct feedback from the instructor during each session, and with a roadmap focused on scanning skills and puncture skills. Additionally, the residents’ improvement in the simulated environment was transferred to a real patient situation. It should be emphasized that real clinical conditions are always different and more challenging than a simulated environment, where every training session has the same conditions in a protected environment.

It is difficult to compare our results with previously published learning curves for regional anesthesia procedures since previous studies were performed to teach different skills. Barrington et al. determined the learning curve for acquiring “sonographic skills” for an axillary brachial plexus block in volunteer models [3]. They concluded that novices require 10 hands-on training sessions (median duration of 5 min, with a 1 to 15 min range) to acquire sonographic proficiency. Similarly, Areti et al. determined the learning curve of “scanning skills”, in anesthesiology residents who scanned volunteers to identify structures of the brachial plexus above the clavicle. They concluded that residents required seven scanning sessions to identify the aimed structures properly [11]. On the other hand, Kim et al. described learning curves for “injection skills” when inexperienced students performed injections in a simple phantom made from a piece of spaghetti to simulate a nerve within a starch core and embedded in elatin. They documented that the number of trials necessary for a satisfying ultrasound-guided injection was 5 [10]. Finally, Kollmann et al. described students’ learning curves of CUSUM method applied to ultrasound-guided continuous femoral nerve block on real patients and concluded that students needed 12 attempts to master the technique [12]. There are many differences between our study and the studies described above in terms of the skills trained (scanning skills, injection skills, versus catheter insertion), the simulation model used (low-fidelity phantoms versus real patient settings), the block performed (brachial plexus versus femoral versus popliteal), and the duration of the intervention (time for training). Because of this, at this point, there is not enough evidence to establish a standardized recommendation of numbers of simulated sessions needed to teach regional anesthesia blocks.

We want to highlight the relevance of the instructional model involved in a simulation-based education and training. Duration of training seems to be very relevant. For example, Barrington et al. documented that proficiency of participants in capturing sonograms and identifying anatomical structures relevant for axillary brachial plexus requires 2.75 h. [3]. Nevertheless, there are many other factors that might determine students’ skill acquisition such us number of sessions, distribution of the practice (massive versus distributed), type of feedback and degree of instructor’s training. As educators, when implementing a simulation-based training program in our curriculum, we should carefully analyze the instructional model used in a study. Finally, as researchers, it is important to describe in detail all the training conditions we used to standardize training in the future.

Regarding the use of the tracking motion device ICSAD, it has demonstrated construct validity in many surgical procedures, including open, laparoscopic, and microsurgery [19]. More recently, they have been used in anesthesia as assessment tools for procedural skills, demonstrating its construct and concurrent validity in labor epidural placement, spinal anesthesia, ultrasound-guided supraclavicular block, and jugular CVC placement [6, 14, 20, 21]. Three dexterity scores can be measured: total distance traveled by each hand, number of movements, and total time of the procedure. Notably, the dexterity number of movements is determined based on a calibration process of translational and rotational velocity thresholds. Therefore, the number of movements registered is highly dependent upon the thresholds the researchers have pre-defined, so careful interpretation should be considered with this number. In the present study, ICSAD dexterities TPL and NM were not significant when comparing the first with the last session. A lack of power might explain this situation because the sample size was calculated for the primary outcome (GRS scores).

The concept of transference refers to the fact that skills acquired through simulation-based training may be applied by students on real patients in clinical settings. In the past, transfer studies used a control group (no simulation-based training) to have a comparison with the simulation trained group [13]. Nowadays, the evidence and the state of art of simulation hinder the development of protocols with a no simulation control group [4]. In this context, and accordingly to the definition of transference, the comparison in this study was done between residents’ final training session and the application of said training on a real patient [22, 23].

This study documents the transference of residents’ skill improvement in a simulated environment to a real patient situation. Residents’ performance was maintained when skills were transferred from the simulated model to the real patient situation. Median values of GRS scores decrease slightly from 28.3 to 26.5 points. Additionally, our results show that residents needed more time for the first block in a clinical situation compared to the last simulated session. The cognitive load may explain this difference in performing the first block on a real patient, combined with the challenge of a different anatomical situation. According to the following studies, the factors affecting the transfer of a skill are classified into 3 categories: learner characteristics, training design, and work environment [24]. Transfer of learning should be the endpoint of simulation-based training, but unfortunately it is not an automatic and direct outcome, [25] and little interest was placed in this process. In a systematic review about ultrasound-guided regional anesthesia (UGRA) simulation training, [26] only one study reported that the acquired UGRA skills may be transferred to the clinical setting [8].

Our study has several limitations. First, there is a need for more power to find differences in TPL in meters when comparing the first simulated session with the last. As explained above, the sample size was calculated to find a difference in GRS scores based on previous studies using this observational scale. Additionally, the sample size was limited by the number of residents per year of our program and by the complexity of logistics when training and assessing ten residents in a 10-h training program. Another possible explanation could be that in a single shot block, hand movements are restricted to a small area, impeding the measurement of different total path length. Second, we designed a structured 10-h training program to assess the progression of skills over time in novice residents with no prior ultrasound experience. However, implementing such extensive training may be challenging for anesthesia educators due to resource constraints. A more practical approach, informed by the findings of this study, would be to tailor training to the learners’ initial skill levels and to deliver a reasonable number of sessions. Based on our results, programs comprising approximately 4 to 6 focused sessions may be sufficient to develop core competencies in peripheral nerve block techniques. Third, another limitation is the lack of control of all the variables during the final assessment in the PACU unit with a real patient. For example, anatomical variations between patients, calm patients versus patients in severe pain, may affect the flow of resident’s performance, impacting the total time needed to perform the procedure. Fourth, transference reported in this study cannot be attributed to the simulation training alone because residents during the 10 weeks of training continue to be exposed to real-life cases that can improve their overall procedural skills. Another limitation of this type of studies is the subjectivity of the assessment method, even if the inter-rater reliability for the observers was good. Finally, the lack of a control group prevents us from comparing our results with standard approaches.

## Conclusions

This study demonstrated that this simulation-based training program significantly improves anesthesia first-year residents’ proficiency in a single-shot ultrasound-guided popliteal nerve block. This improvement was transferred to a real patient setting. The learning curve plateaued at session 7, this could be interesting information when designing training programs in regional anesthesia for single shot blocks, taking in consideration the instructional methodology applied in this study. Nowadays, the implementation of this type of programs before performing blocks on real patients, may have a contribution to patient safety.

## Data Availability

The datasets used and/or analyzed during the current study are available from the corresponding author on reasonable request.

## Abbreviations

GRS: Global rating scale
ICSAD: Imperial College Surgical Assessment Device
TPL: Total path length
NM: Number of movements
TPT: Total procedure time
PNB: Peripheral nerve block
PACU: Post anesthesia care unit
ICC: Intraclass correlation coefficient
SBT: Simulation-based training
